# The genetic and molecular basis for improving heat stress tolerance in wheat

**DOI:** 10.1007/s42994-021-00064-z

**Published:** 2021-12-03

**Authors:** Lv Sun, Jingjing Wen, Huiru Peng, Yingyin Yao, Zhaorong Hu, Zhongfu Ni, Qixin Sun, Mingming Xin

**Affiliations:** Key Laboratory of Crop Heterosis Utilization (MOE), China Agricultural University, Beijing, 100193 China

**Keywords:** Wheat, Heat stress, Genetic basis, Molecular mechanisms

## Abstract

Wheat production requires at least ~ 2.4% increase per year rate by 2050 globally to meet food demands. However, heat stress results in serious yield loss of wheat worldwide. Correspondingly, wheat has evolved genetic basis and molecular mechanisms to protect themselves from heat-induced damage. Thus, it is very urgent to understand the underlying genetic basis and molecular mechanisms responsive to elevated temperatures to provide important strategies for heat-tolerant varieties breeding. In this review, we focused on the impact of heat stress on morphology variation at adult stage in wheat breeding programs. We also summarize the recent studies of genetic and molecular factors regulating heat tolerance, including identification of heat stress tolerance related QTLs/genes, and the regulation pathway in response to heat stress. In addition, we discuss the potential ways to improve heat tolerance by developing new technologies such as genome editing. This review of wheat responses to heat stress may shed light on the understanding heat-responsive mechanisms, although the regulatory network of heat tolerance is still ambiguous in wheat.

**Supplementary Information:**

The online version contains supplementary material available at 10.1007/s42994-021-00064-z.

## Introduction

Wheat (*Triticum aestivum* L.) is the most widely grown staple crop in the world, cultivated from 67° N in Scandinavia and Russia to 45° S in Argentina. It serves as a rich source of proteins, minerals and other essential nutrients for approximately 30% of the human population (IWGSC 2014). Due to the increasing population, wheat production requires ~ 2.4% increase per year to meet global food demands by 2050 (Ray et al. 2013). As a chimonophilous plant, wheat is sensitive to heat stress and prefers an optimal daytime growing temperature of 20–24 °C during reproductive development (Farooq et al. 2011). Model predictions indicate that global wheat production will fall by 6% per 1 °C increase above optimum temperature (Asseng et al. 2015). Since the Industrial Revolution, the average global surface temperature has warmed by 0.85 °C (IWGSC 2014), and this trend will continue and is expected to rise more than 1.5 °C by the end of twenty-first century (Wheeler and Braun 2013). According to the simulation analysis, the average wheat yield decreased by 1–28% during 1981–2010 period caused by rising temperature (Asseng et al. 2015). Thus, the warming temperature causes severe wheat yield loss and imposes a substantial risk to global food security. To cope with climate variations and to protect themselves from injury and damage, wheat has evolved complex systems to improve their capability in response to heat stress. Therefore, understanding the molecular and genetic basis of the wheat response to heat stress would be helpful to develop new strategies to minimize deleterious impacts of heat stress during wheat breeding programs.

## Phenotypic variation in reproductive stage responsive to heat stress in wheat

Heat stress imposes diverse negative effects on agronomic traits at different wheat developmental stages, but pre-flowering and anthesis stages are expected to be the most sensitive stages to heat stress (Cossani and Reynolds 2012), since unexpected high temperature could reduce pollen viability and subsequently decrease grain number, grain filling and grain quality (Asseng et al. 2011; Ugarte et al. 2007). It is reported that wheat pollen viability and seed setting rate will decrease significantly when the high temperature (> 30 °C) appears at the anthesis stage (Browne et al. 2021; Djanaguiraman et al. 2020). Consistently, a five-day period with moderate high temperate (~ 24 °C) at beginning of the heading period can reduce floret fertility by 15%, whereas extreme high temperature (~ 35 °C) will lead to complete abortion (Prasad and Djanaguiraman 2014). Not surprisingly, daytime high temperature (34 °C) at the anthesis stage significantly decreased wheat seed set from 7 to 19% (Sun et al. 2018). In addition, nighttime high temperature possesses similar effects to seed set rate, and 7-day-long high temperature at night (24 °C) in anthesis period result in decreased seed set by 15% in wheat (Narayanan et al. 2015).

Besides grain number, seed size and thousand kernel weight were also adversely affected by heat stress. Although high temperature can accelerate grain filling rate to some extent (Asseng et al. 2015; Barlow et al. 2015; Lobell et al. 2012), it shortens grain filling duration by 0.30–0.60% for every unit increase of high-temperature days when temperature exceed 30 °C (Liu et al. 2016). Bella and their colleagues reported that the duration and the timing of heat stress can explain 51.6% of phenotypic variation of thousand-kernel weight by analyzing more than 100 wheat varieties with varied geographic origins (Balla et al. 2019). Wang et al. (2018) found that late sowing can cause an increase of ~ 2 °C during the wheat filling stage and reduced the grain filling duration by 1–2 weeks, finally resulted in a substantial yield decrease. Bheemanahalli et al. (2019) examined daytime heat response of 28 spring wheat varieties during flowering and grain filling stage, and found ~ 32 and ~ 16% decrease of thousand kernel weight of main spike, respectively. Similarly, nighttime high temperature at post-anthesis stage also reduced wheat thousand-grain weight by ~ 3% per °C increase (García et al. 2016). Moreover, other studies confirmed these observations both in field and in greenhouse (Liu et al. 2020; Talukder et al. 2014a).

As we know, starch contributes about ~ 80% of the dry weight of wheat seed, which has a close link with wheat grain yield. Liu et al. (2011) applied 3-day period heat stress to wheat at the different filling stage from 1 to 33 days after flowering, and found different effects of heat stress at different periods of grain filling on grain starch formation of wheat. The effect of heat treatment at an early stage (6–8 days after flowering) is greater than that at late stage (36–38 days after flowering). Further investigation showed heat stress reduced both amylose and amylopectin concentration, yet amylopectin accumulation is more sensitive to the stress than that of amylose (Liu et al. 2011). Consistent with the observation, the expression patterns of starch biosynthesis-related genes changed seriously in response to heat stress, e.g. ADP-glucose pyrophosphorylase, one of the key enzymes during starch biosynthesis, was down-regulated after heat stress together with other related genes, and directly associated with the decrease of starch accumulation (Hurkman et al. 2003).

## Genetic basis in response to heat stress in wheat

Heat stress tolerance is a quantitative trait contributed by many minor QTLs (Bohnert et al. 2006), and it is more difficult to measure phenotypic variation in response to heat stress compared with other agronomic traits. Therefore, there is very limited available information about the genetic basis of heat stress response in wheat, and none heat-tolerance gene was isolated according to map-based cloning strategy by now. Yet, many studies have been trying to map genetic loci controlling heat stress tolerance in wheat. In 1990’s, Sun and Quick reported that chromosomes 3A, 3B, 4A, 4B and 5A contained heat stress-tolerance related loci in tetraploid wheat because their corresponding chromosome substitution lines showed impaired heat tolerance by measuring membrane thermal stability (Sun and Quick 1991). Later, Sun’s group further confirmed the observation and found chromosomes 3A and 3B associated with heat tolerance in wheat cultivar Hope (Xu et al. 1996). In the twenty-first century, increasing heat stress-tolerance related QTL loci were reported taking advantage of developing molecular marker technology. Yang and the colleagues generated an F_2_ population including 166 individuals using heat-tolerant cultivar Ventnor and heat-susceptible cultivar Karl92, and identified two QTLs controlling grain-filling duration in response to heat stress on chromosomal 1B and 5A, which linked to the simple sequence repeat marker Xgwm11 and Xgwm293, respectively (Yang et al. 2002). Using a similar heat treatment to Yang’s method, Mohammadi et al. (2008) detected three heat-tolerance QTLs on chromosomes 1B, 5B and 7B in terms of heat susceptibility index (HSI, an indicator of heat response) by examining 144 recombinant inbred lines (RILs) with varied heat sensitivities derived from Kauz and MTRWA116 cultivar. Later, Mason et al. (2010) analyzed the HSI of yield component of a Halberd (heat tolerant)/Cutter (heat susceptible) RIL population under controlled heat stress environments (38 °C day/18 °C night), and detected 27 QTLs associated with improved heat tolerance, and among which, five (located on chromosomes 1A, 2A, 2B and 3B) were simultaneously detected in two-year experiments. Moreover, a follow-up study by the same group mapped 14 QTLs contributing to heat tolerance in wheat by calculating HSI of kernel number, total kernel weight, and single kernel weight coupled with temperature depression of the main spike and main flag leaf. Of these genomic loci, seven regions were consistently detected in their two continuous studies. Each QTL explains approximately 4.5–19.3% phenotypic variance, and a combination of the superior haplotype of three QTLs on chromosomes 1B, 5A, and 6D can improve the genetic effect of heat tolerance compared with a single locus (Mason et al. 2011). Pinto et al. (2010) also identified 16 QTLs associated with heat stress adaptive traits using Seri/Babax RIL population, and a QTL located on 4A explained 17% phenotypic variation under heat stress conditions. Interestingly, six common QTLs were found to contribute to both heat and drought stress tolerance, indicating a crosstalk between two stresses (Pinto et al. 2010). Paliwal et al. (2012) identified two heat tolerance QTLs on chromosomes 2B and 7B by analyzing HSI of 1000-grain weight, grain fill duration and canopy temperature of 144 wheat RIL lines, which explained phenotypic variation ranging from 9.78 to 20.34%. Sangwan et al. (2019) created a RIL population of wheat (*Triticum aestivum* L.) with heat-tolerant parent WH1021 and heat-sensitive parent WH711, significant genomic regions associated with heat tolerance were detected on chromosomes 2A, 2D, 4A and 5A, and a consistent QTL was found on chromosome 2D based on photosynthetic rate analysis. Zhai et al. (2021) located a *TaHST1* locus in an interval of 0.949 Mbp at the distal terminus of 4AL chromosome arm, which contained 19 high confidence genes and contributed to both vegetative and reproductive growth of wheat under heat stress conditions. Moreover, genome-wide association analysis (GWAS) was also exploited to detect heat responsive QTLs using 205 wheat varieties with a late sown method, and a total of 69 potential QTLs were identified for ten different traits including grain filling duration and grain filling rate (Kumar et al. 2020). In addition, Wang et al. (2021) performed GWAS analysis of 688 diverse winter wheat accessions on thousand-grain weight and stress susceptibility index in response to heat stress using 90 K array, and revealed that terminal heat stress tolerance is not improved concurrently with grain weight during wheat breeding programs during recent decades, the authors proved superior alleles regulating both grain weight and heat tolerance, which can be used in marker-assisted selection for wheat in future. We summarized the reported QTLs-related heat response in wheat in Table 1 and Table S1.

**Table 1:** Summary of heat stress-related QTLs in wheat

Trait	QTL	Marker or flanking marker	Chromosomes	Confident interval (cM) or (bp)	LOD	*R*^2^	
HSI of Kernel number of main-spike	QHknm.tam-1A	cfa2129	1A	–	3.40	0.274	Mason et al. (2010)
HSI of Thousand grain weight	QHSI oftgw.cau-1A	RAC875_c54380_249	1A	78.2 cM	4.87	0.077	Guan et al. (2018)
Grain number per main spike	QGNP-HS-R1	AX-95652063-AX-95660318	1A	98.3–99.2 cM	20.41	0.245	Li et al. (2019)
Grain yield per plant	QGYP-HS-R1	AX-111105973-AX-94402739	1A	81.2–82.5 cM	13.50	0.210	Li et al. (2019)
Grain-filling duration	–	Xgwm11	1B	–	–	0.120	Yang et al. (2002)
SSI of Kernel weight	–	gwm190	1B	0–14 cM	3.43	0.443	Mohammadi et al. (2008)
HSI of Kernel weight of main-spike	QHkwm.tam-1B	gwm268	1B	–	2.90	0.106	Mason et al. (2010)
HSI of Kernel weight of main-spike	QHkwm.tam-1B	gwm153	1B	–	3.93	0.101	Mason et al. (2011)
SPAD chlorophyll content	QHtscc.ksu-1B	gwm18, Bin1130	1B	2.3 cM	2.50	0.114	Talukder et al. (2014b)
Thousand kernel weight	QTKW-HS-R1	AX-94745844-AX-110935476	1D	111.6–126 cM	2.58	0.042	Li et al. (2019)
Spikelet number per spike	QSpn.agt-SG.1D	–	1D	23.4–24.6 cM	3.41	0.062	Telfer et al. (2021)
Thylakoid membrane damage	QHttmd.ksu-1D	Bin747, Bin1596	1D	5.31 cM	3.06	0.141	Talukder et al. (2014b)
HSI of Single kernel weight of main-spike	QHskm.tam-2A	gwm294	2A	–	3.70	0.178	Mason et al. (2010)
HSI of Single kernel weight of main-spike	QHskm.tam-2A c	gwm356	2A	–	2.40	0.210	Mason et al. (2010)
Plant heigh	Qph.ccshau-2A	xgwm512-xgwm448	2A	35.3 cM	2.10	0.177	Sangwan et al. (2019)
Days to heading	Qdh.ccshau-2A	xgwm512-xgwm448	2A	35.3 cM	2.70	0.061	Sangwan et al. (2019)
iWUE	Qiwu.ccshau-2.1A	xgwm497-xgwm512	2A	5.2 cM	2.70	0.005	Sangwan et al. (2019)
iWUE	Qiwu.ccshau-2.2A	xgwm512-xgwm448	2A	35.3 cM	3.60	0.389	Sangwan et al. (2019)
iWUE	Qiwu.ccshau-2.3A	xgwm512-xgwm448	2A	35.3 cM	10.00	0.480	Sangwan et al. (2019)
SSI of Grain yeild	QTL.ICD.Heat.03	AX-94538070	2A	748624588 bp	3.06	0.250	Hassouni et al. (2019)
HSI of Thousand Grain Weight	Qtgw.iiwbr-2A	Gwm122	2A	171.41 cM	12.17	0.199	Bhusal et al. (2017)
HSI of Grain weight/ main spike	Qgws.iiwbr-2A	GWM448	2A	170.01 cM	4.45	0.756	Bhusal et al. (2017)
Grain yield per spike	QGwe.agt-RG.2A	–	2A	69–71.1 cM	2.76	0.123	Telfer et al. (2021)
Number of leaves per seedling	QLNHR.nri-2A.2	IWB61157	2A	150.11 cM	–	0.083	Maulana et al. (2018)
HSI of Kernel number of main-spike	QHknm.tam-2B	gwm111.2	2B	–	3.60	0.127	Mason et al. (2010)
HSI of Kernel number of main-spike	QHknm.tam-2B	barc200.2	2B	–	3.40	0.216	Mason et al. (2010)
HSI of thousand grain weight	QHtHSI oftgw.bhu—2B	Xgwm935 – Xgwm1273	2B	23 cM	3.40	0.178	Paliwal et al. (2012)
Grain number per main spike	QGNP-HS-R2	AX-109501025-AX-108731558	2B	58–61.1 cM	7.94	0.078	Li et al. (2019)
Grain yield per plant	QGYP-HS-R2	AX-94940181-AX-108730045	2B	53.1–54.9 cM	8.84	0.126	Li et al. (2019)
HSI of grain filling duration	QhtHSI ofgfd.iiwbr-2B	Gwm257	2B	28.01 cM	7.38	0.128	Bhusal et al. (2017)
Leaf chlorophyll content	QLCCHR.nri-2B	IWB55435	2B	27.2 cM	–	0.068	Maulana et al. (2018)
Plasma membrane damage	QHtpmd.ksu-2B	Bin178, Bin81	2B	6.47 cM	3.75	0.172	Talukder et al. (2014b)
Grain yield	QGY-2B	aag/ctc-13-acc/ctc-9	2B	91.1 cM	–	0.101	Hassan et al. (2018)
HSI of Single kernel weight of main-spike	QHskm.tam-2D.1	gwm261	2D	–	11.77	0.193	Mason et al. (2011)
HSI of single kernel weight of main-spike	QHskm.tam-2D.2	cfd56	2D	–	3.61	0.052	Mason et al. (2011)
Days to heading	Qdh.ccshau-2D	barc124-xgwm102	2D	27.9 cM	4.20	0.007	Sangwan et al. (2019)
Days to maturity	Qdm.ccshau-2.1D	barc124-xgwm102	2D	27.9 cM	3.40	0.015	Sangwan et al. (2019)
Days to maturity	Qdm.ccshau-2.2D	gwm249-gwm382	2D	25.6 cM	2.74	0.084	Sangwan et al. (2019)
Photosynthetic rate (Pn)	Qpn.ccshau-2.1D	barc124-xgwm102	2D	27.9 cM	3.60	0.781	Sangwan et al. (2019)
Photosynthetic rate (Pn)	Qpn.ccshau-2.2D	barc124- xgwm102	2D	27.9 cM	3.40	0.050	Sangwan et al. (2019)
HSI of thousand grain weight	QHSI oftgw.cau-2D	Kukri_c19540_425	2D	90 cM	7.58	0.143	Guan et al. (2018)
Leaf chlorophyll content	QLCCHR.nri-2D.1	IWB18745	2D	22.46 cM	–	0.058	Maulana et al. (2018)
Leaf chlorophyll content	QLCCHR.nri-2D.2	IWB66401-IWB36817	2D	70.65–85.97 cM	–	0.188	Maulana et al. (2018)
Grain yield	QGY-2D	wPt-6657-gdm035	2D	4 cM	–	0.116	Hassan et al. (2018)
Grain number per main spike	QGNP-HS-R3	AX-94684189-AX-110122723	3A	0–0.8 cM	4.15	0.038	Li et al. (2019)
Grain number per main spike	QGNP-HS-R4	AX-111656976-AX-110051593	3A	180.3–185.7 cM	3.01	0.027	Li et al. (2019)
Grain yield per plant	QGYP-HS-R3	AX-111659209-AX-94667190	3A	212.4–217.1 cM	3.21	0.042	Li et al. (2019)
Grain yield per plant	QGYP-HS-R4	Xwmc532-AX-109285546	3A	43.3–49.5 cM	3.54	0.047	Li et al. (2019)
Spikelet number per spike	QSpn.agt-RG.3A.1	–	3A	58.1–58.8 cM	2.82	0.070	Telfer et al. (2021)
Number of leaves per seedling	QLNHR.nri-3A	IWB50704	3A	177.24 cM	–	0.067	Maulana et al. (2018)
HSI of Kernel weight of main-spike	QHkwm.tam-3B	wmc326	3B	–	5.40	0.212	Mason et al. (2010)
HSI of Kernel weight of main-spike	QHkwm.tam-3B	wmc527	3B	–	4.80	0.190	Mason et al. (2010)
HSI of Kernel number of main-spike	QHknm.tam-3B c	barc147	3B	–	2.70	0.113	Mason et al. (2010)
HSI of Single kernel weight of main-spike	QHskm.tam-3B	barc229	3B	–	3.17	0.045	Mason et al. (2011)
HSI of single grain weight	QHsgw.aww-3B	wsnp_BE497169B_Ta_2_1	3B	3.2 cM	8.10	0.201	Shirdelmoghanloo et al. (2016)
HSI of single grain weight	QHsgw.aww-3B	wsnp_Ex_c12875_20407926	3B	1.4 cM	4.70	0.108	Shirdelmoghanloo et al. (2016)
Shoot length	QSLHR.nri-3B.1	IWB1428	3B	9.7 cM	–	0.062	Maulana et al. (2018)
Shoot length	QSLHR.nri-3B.2	IWB26717	3B	67.17 cM	–	0.056	Maulana et al. (2018)
HSI of Kernel number of main-spike	QHknm.tam-4A	wmc89	4A	–	4.60	0.155	Mason et al. (2010)
HIS Single kernel weight of main-spike	QHskm.tam-4A	barc170	4A	–	4.60	0.135	Mason et al. (2010)
HSI of Single kernel weight of main-spike	QHskm.tam-4A.1	wmc707	4A	–	5.50	0.096	Mason et al. (2011)
HSI of Single kernel weight of main-spike	QHskm.tam-4A.2	wmc313	4A	–	7.55	0.123	Mason et al. (2011)
Plant height	Qph.ccshau-4A	xgwm165-xcfd71	4A	25.1 cM	3.20	0.334	Sangwan et al. (2019)
iWUE	Qiwu.ccshau-4A	xgwm165-xcfd71	4A	25.1 cM	3.70	0.007	Sangwan et al. (2019)
Transpiration rate (E)	Qe.ccshau-4A	xgwm165-xcfd71	4A	25.1 cM	3.60	0.063	Sangwan et al. (2019)
Chlorophyll fluorescence (Fv/Fm)	TaHST1	Xhau-1	4A	chr4A:743,680,033–743680857 bp	–	–	Zhai et al. (2021)
Chlorophyll fluorescence (Fv/Fm)	TaHST1	Xhau-2	4A	chr4A:743,680,939–743681364 bp	–	–	Zhai et al. (2021)
Chlorophyll fluorescence (Fv/Fm)	TaHST1	Xhau-3	4A	chr4A:744,277,785–744278572 bp	–	–	Zhai et al. (2021)
Chlorophyll fluorescence (Fv/Fm)	TaHST1	Xhau-4	4A	chr4A:744,311,304–744311430 bp	–	–	Zhai et al. (2021)
Chlorophyll fluorescence (Fv/Fm)	TaHST1	Xhau-5	4A	chr4A:744,530,489–744530747 bp	–	–	Zhai et al. (2021)
Leaf chlorophyll content	QLCCHR.nri-4A	IWB37183	4A	8.61 cM	–	0.066	Maulana et al. (2018)
Water soluble carbohydrates	QWSC-4A	act/cag-3-agg/cta-12	4A	13.1 cM	–	0.105	Hassan et al. (2018)
HSI of thousand grain weight	QHSI oftgw.cau-4B.1	Excalibur_c51845_186	4B	111 cM	3.24	0.054	Guan et al. (2018)
HSI of thousand grain weight	QHSI oftgw.cau-4B.2	WMC652	4B	119.1 cM	5.26	0.092	Guan et al. (2018)
HSI of thousand grain weight	QHSI oftgw.cau-4B.2	gpw7390	4B	120.8 cM	6.21	0.099	Guan et al. (2018)
SSI of thousand grain weight	QSsi.cau.4B_33	wsnp_Ex_c18318_27140346-wsnp_Ra_c9755_16200944	4B	33 cM	–	0.023–0.024	Wang et al. (2021)
Spikelet number per spike	QSpn.agt-RG.4B	–	4B	80.9–82.2 cM	2.96	0.065	Telfer et al. (2021)
Leaf chlorophyll content	QLCCHR.nri-4B.1	IWB48055	4B	39.93–41.65 cM	–	0.066	Maulana et al. (2018)
Leaf chlorophyll content	QLCCHR.nri-4B.2	IWB42264-IWB35851	4B	75.65 cM	–	0.185	Maulana et al. (2018)
Number of leaves per seedling	QLNHR.nri-4B	IWB64397-IWB10366	4B	68.45–71.46 cM	–	0.131	Maulana et al. (2018)
Cytoplasmic membrane stability	QCMS-4B	wPt-1708-wmc048a	4B	9.3 cM	–	0.100	Hassan et al. (2018)
Thousand kernel weight	QTKW-HS-R2	AX-111475478-AX-89654830	4D	4.9–10.5 cM	2.83	0.046	Li et al. (2019)
Spikelet number per spike	QSpn.agt-RG.4D	–	4D	47.8–46.2 cM	2.61	0.056	Telfer et al. (2021)
Grain-filling duration	–	Xgwm293	5A	–	–	0.110	Yang et al. (2002)
HSI of Kernel weight of main-spike	QHkwm.tam-5A	gwm291	5A	–	3.50	0.219	Mason et al. (2010)
HIS Single kernel weight of main-spike	QHskm.tam-5A	barc151	5A	–	3.00	0.098	Mason et al. (2010)
HSI of Kernel number of main-spike	QHknm.tam-5A.1	barc197	5A	–	3.50	0.138	Mason et al. (2010)
HSI of Kernel number of main-spike	QHknm.tam-5A.2	gwm126	5A	–	3.80	0.321	Mason et al. (2010)
HSI of Kernel weight of main-spike	QHkwm.tam-5A.1	gwm179	5A	–	3.95	0.122	Mason et al. (2011)
HSI of Kernel weight of main-spike	QHkwm.tam-5A.2	gwm291	5A	–	3.81	0.114	Mason et al. (2011)
HSI of Single kernel weight of main-spike	QHskm.tam-5A	gwm443	5A	–	4.04	0.058	Mason et al. (2011)
NDVI	Qndvi.ccshau-5A	barc186-barc141	5A	28.8 cM	2.20	0.083	Sangwan et al. (2019)
SSI of Grain yeild	QTL.ICD.Heat.08§	AX-94631521	5A	421078546 bp	4.93	0.450	Hassouni et al. (2019)
Thousand kernel weight	QTKW-HS-R3	AX-111764369-AX-95659703	5A	62–63.4 cM	10.59	0.195	Li et al. (2019)
Grain yield per plant	QGYP-HS-R5	AX-95630862-AX-95630256	5A	52.9–57 cM	2.88	0.038	Li et al. (2019)
SSI of thousand grain weight	QSsi.cau.5A_91	IAAV3365-Kukri_c33022_198	5A	91 cM	–	0.351–0.473	Wang et al. (2021)
Spikelet number per spike	QSpn.agt-RG.5A.3	–	5A	154.8–162.5 cM	3.46	0.090	Telfer et al. (2021)
SSI of Kernel weight	–	gwm133A	5B	112–132 cM	2.01	0.273	Mohammadi et al. (2008)
HSI of Kernel number of main-spike	QHknm.tam-5B	gwm213	5B	–	5.70	0.246	Mason et al. (2010)
HSI of single kernel weight of main-spike	QHskm.tam-5B	wmc73	5B	–	4.08	0.062	Mason et al. (2011)
HSI of Kernel number of main-spike	QHknm.tam-5B	gwm408	5B	–	3.05	0.134	Mason et al. (2011)
HSI of thousand grain weight	QHSI oftgw.cau-5B	barc59	5B	81.9 cM	3.15	0.048	Guan et al. (2018)
SSI of grain yield	QTL.ICD.Heat.09§	AX-95182463	5B	427098066 bp	4.17	0.370	Hassouni et al. (2019)
Grain number per main spike	QGNP-HS-R5	AX-95658487-AX-109829036	5B	173–188.7 cM	8.25	0.081	Li et al. (2019)
Leaf chlorophyll content	QLCCHR.nri-5B	IWB64287-IWA4329	5B	182.15–188.58 cM	–	0.249	Maulana et al. (2018)
Number of leaves per seedling	QLNHR.nri-5B.1	IWB71913-IWB43528	5B	49.02 cM	–	0.129	Maulana et al. (2018)
Number of leaves per seedling	QLNHR.nri-5B.2	IWB58120	5B	144.26 cM	–	0.059	Maulana et al. (2018)
Proline content	QPro-5B	acc/ctc-3-gwm133	5B	7.0 cM	–	0.124	Hassan et al. (2018)
Thousand Kernel weight	QTKW-HS-R4	AX-108805055-AX-109308225	5D	138.3–142.6 cM	3.33	0.055	Li et al. (2019)
SSI of Thousand grain weight	QSsi.cau.5D_138	RFL_Contig1091_1538	5D	138 cM	–	0.030	Wang et al. (2021)
HSI of Thousand grain weight	QHSI oftgw.cau-6A	BS00068092_51	6A	32.8 cM	2.93	0.065	Guan et al. (2018)
Spikelet number per spike	QSpn.agt-RG.6A.1	–	6A	14.9–18.7 cM	4.27	0.113	Telfer et al. (2021)
Spikelet number per spike	QSpn.agt-RG.6A.2	–	6A	125.1–126 cM	4.71	0.111	Telfer et al. (2021)
Thylakoid membrane damage	QHttmd.ksu-6A	Xbarc113, AGCTCG347	6A	6.98 cM	2.58	0.119	Talukder et al. (2014b)
Maximum efficiency of photosystem II (Fv/Fm)	QFv/Fm-6A	wmc0256-acc/ctg-6	6A	68.8 cM	–	0.112	Hassan et al. (2018)
HSI of thousand grain weight	QHSI oftgw.cau-6B	BS00009825_51	6B	13 cM	5.40	0.093	Guan et al. (2018)
SSI of grain yield	QTL.ICD.Heat.10§	AX-94408589	6B	157777006 bp	3.20	0.360	Hassouni et al. (2019)
Grain number per main spike	QGNP-HS-R6	AX-95177681-AX-94427873	6B	87.4–87.5 cM	17.52	0.200	Li et al. (2019)
Thousand Kernel weight	QTKW-HS-R5	AX-110986080-AX-109476271	6B	97.6–98.1 cM	3.74	0.064	Li et al. (2019)
HSI of single grain weight	QHsgw.aww-6B	wsnp_Ex_c11573_18650189	6B	9.1 cM	3.80	0.121	Shirdelmoghanloo et al. (2016)
Spikelet number per spike	QSpn.agt-RG.6B	–	6B	17.8–19.2 cM	3.20	0.072	Telfer et al. (2021)
HSI of single kernel weight of main-spike	QHskm.tam-6D	cfd49	6D	–	6.01	0.147	Mason et al. (2011)
HSI OF thousand Grain weight	QHSI oftgw.cau-6D	IACX10982	6D	134.8 cM	4.73	0.075	Guan et al. (2018)
HIS single kernel weight of main-spike	QHskm.tam-7A	gwm282	7A	–	4.30	0.316	Mason et al. (2010)
HSI of Single kernel weight of main-spike	QHskm.tam-7A	wmc603	7A	–	4.27	0.093	Mason et al. (2011)
Thylakoid membrane damage	QHttmd.ksu-7A	Xbarc121, barc49	7A	11.12 cM	4.15	0.192	Talukder et al. (2014b)
SPAD chlorophyll content	QHtscc.ksu-7A	Bin754, Bin45	7A	3.72 cM	4.22	0.195	Talukder et al. (2014b)
SSI of Kernel weight	–	gwm63B	7B	68–86 cM	2.61	0.340	Mohammadi et al. (2008)
HSI of Single kernel weight of main-spike	QHskm.tam-7B	wmc182	7B	–	3.79	0.055	Mason et al. (2011)
Number of leaves per seedling	QLNHR.nri-7B	IWB34893	7B	145.29 cM	–	0.061	Maulana et al. (2018)
HSI of thousand grain weight	QHtHSI oftgw.bhu—7B	Xgwm1025 – Xgwm745	7D	3.6 cM	8.70	0.203	Paliwal et al. (2012)
HSI of thousand grain weight	QHtHSI oftgw.bhu—7D	Xgwm1025 – Xgwm745	7D	3.1 cM	3.50	0.098	Paliwal et al. (2012)
Shoot length	QSLHR.nri-7D	IWB12476, IWB12582	7D	26.92 cM	–	0.126	Maulana et al. (2018)

## Omics-based identification of heat-responsive genes in wheat

Since map-based cloning of the heat tolerance gene of wheat is still difficult in a forward genetic way, reverse genetic methods have been widely used to identify heat-responsive genes in wheat, e.g. multi-omics. Transcriptome analysis including microarray and RNA-seq is recognized as a high-throughput way to detect differentially expressed genes in response to heat stress. Qin and colleagues found that 10.7% probe sets were differentially expressed in response to 40 °C treatment at wheat seedling stage according to microarray analysis, which were involved in phytohormone biosynthesis, calcium and sugar signaling and ribosomal proteins related functional pathways (Qin et al. 2008). Later, Kumar et al. (2015a) identified 1525 heat-responsive genes using RNA-seq analysis, and reported that heat stress disturbed metabolic processes and oxidations-reductions processes in wheat. Moreover, as a typical allohexaploid, bread wheat experienced two independent hybridization and polyploidization events and theoretically contains three homeologs at each genomic loci. Liu et al.’s study revealed thousands of differentially expressed genes under heat stress conditions which exhibited varied time-course expression patterns. Interestingly, ~ 68.4% of homoeologous triplets showed diverse responses to heat stress, which might contribute to enhance thermotolerance in polyploid wheat (Liu et al. 2015).

Besides the transcriptional responses, post-transcriptional regulation also plays an important role in re-organizing transcriptome plasticity and proteomic complexity in response to heat stress. For example, alternative splicing (AS) refers to a RNA processing that multiple transcripts generate from a single gene, which extensively occurs in wheat genome (Yu et al. 2020). Liu and colleagues found that AS occurrence is increased by ~ 40% under heat stress conditions compared to normal conditions, and identified 3576 genes exhibiting AS changes in response to heat stress. It is worth noticing that a subset of homeologous triplets (7.5%) showed altered splicing patterns (Liu et al. 2015, 2018).

In addition, epigenetic modification is also involved in the post-transcriptional regulation of heat response in wheat including DNA methylation and non-coding RNAs. High temperature has a small but significant effect on gene methylation, and approximately 0.1% of genomic loci showed differential DNA methylation in wheat seedlings between 27 and 12 °C conditions. Of these sites, 63% of regions were also differentially expressed in response to elevated temperature, indicating differential methylation is closely associated with expression changes in wheat (Gardiner et al. 2015). Moreover, non-coding RNAs are also reported to participate in regulating heat response in wheat (Kumar et al. 2015b; Ragupathy et al. 2016; Xin et al. 2010). For example, *TamiR159* was downregulated after 2 h heat treatment in heat-sensitive wheat genotype, which targets *TaGAMYB1* and *TaGAMYB2* and directs their cleavage. Overexpression of *TamiR159* in rice caused increased heat sensitivity compared with wild type (Wang et al. 2012). In addition, Xin et al. identified 77 differentially expressed long non-coding RNAs before and after heat stress, parts of which functions probably by generating siRNAs, and interestingly, H_3_K_9_ acetylation is likely associated with long non-coding RNA expression patterns when subjecting to heat stress (Xin et al. 2011).

Wheat responses to heat stress also occur at the translational level. Pioneering studies discovered a set of proteins showing a changed abundance in response to heat stress using two-dimensional electrophoresis and MALDI-TOF–MS methods (Laino et al. 2010; Majoul et al. 2003, 2004; Yang et al. 2011). For example, it is reported that more low molecular weight proteins were produced in the flag leaf of heat–susceptible wheat cultivar than that of heat-tolerant cultivar in response to heat stress (Nandha et al. 2018). Whereas the abundance of proteins in flag leaf related to chlorophyll synthesis, carbon fixation, protein turnover and redox regulation were significantly altered at the grain filling stage (Lu et al. 2017). Furthermore, iTRAQ investigation identified 256 proteins showing differential expression patterns including 126 up-regulated and 130 down-regulated proteins. These proteins were enriched in stimulus response, stress response, kinase activity, and transferase activity categories (Zhang et al. 2017).

## Functional genes in response to heat stress in wheat

Multi-omics studies provide lots of potential candidate genes responsible for heat tolerance, and their molecular functional and signaling pathway analyses further help us to understand underlying mechanisms. Heat shock proteins (HSPs), acting as molecular chaperones assisting correct protein conformation, were induced rapidly in transcriptome analysis under heat stress conditions mostly controlled by heat shock factors. Because the stress can lead to the accumulation of misfolded proteins, and HSPs would help these proteins with correct folding (Vierling 1991). Rampino et al. (2009) reported that the accumulation of HSP transcriptional abundance is proportional to the heat stress duration in durum wheat varieties, and contribute to acquired themo-tolerance. Wheat *TaHSP23.9* was identified as a heat-responsive gene located in the endoplasmic reticulum based on TMT-labeled quantitative proteomic analysis, and its overexpression transgenic *Arabidopsis* exhibited improved heat tolerance (Wang et al. 2020). Heat shock factors (HSFs) also play a central role regulating HSP expression. There are 56 HSF transcription factors in wheat according to the previous prediction, and A2 and A6 type HSF members were highly induced upon heat stress (Xue et al. 2014). Consistently, Bi et al. (2020) demonstrated that ectopic expression of wheat *TaHsfA6f* in *Arabidopsis* resulted in improved tolerance to heat and other abiotic stresses in terms of seedling survival rate (Bi et al. 2020).

**Fig. 1:**
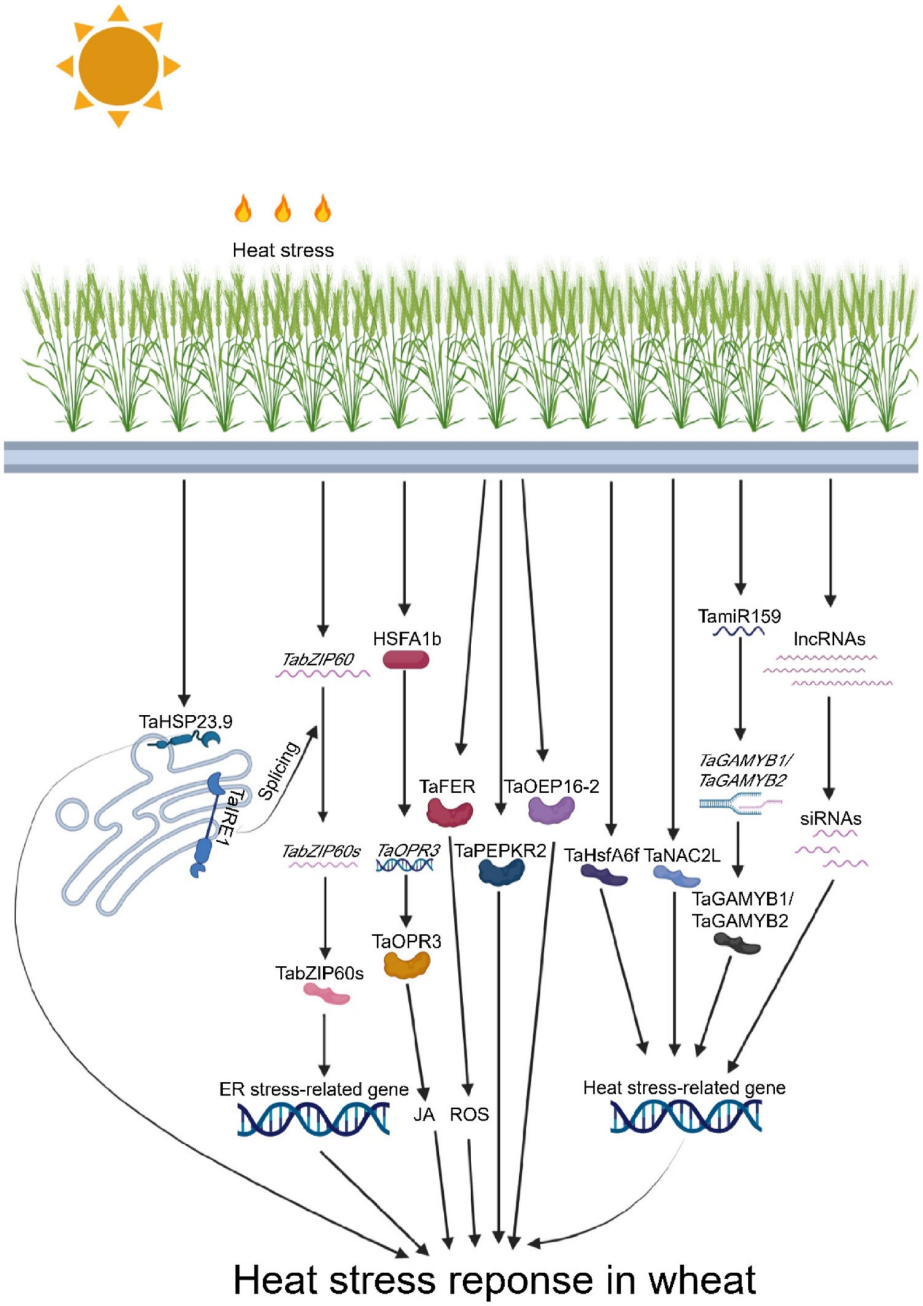
Molecular basis in response to heat stress in wheat

According to the transcriptome analysis, Geng et al. found that *TabZIP60* was up-regulated and subjected to atypical alternative splicing after heat stress, depending on IRE1 gene which recognizes a dual stem-loop structure. Surprisingly, overexpression of heat-induced splicing form of wheat TabZIP60 (TabZIP60s) improved heat tolerance in *Arabidopsis*, but not for the unspliced form. As a transcription factor, TabZIP60s regulates expression patterns of 1104 genes in response to heat stress, including 35 genes, which significantly enriched in ER stress-related GO categories (Geng et al. 2018). In addition, Zang et al. found that *TaFER* (ferritin protein), *TaPEPKR2* (phosphoenolpyruvate carboxylase kinase-related kinase protein), and TaOEP16-2 (plastid outer envelope protein) identified from heat stress-responsive transcriptome analysis, contributing to heat tolerance by overexpression analysis in *Arabidopsis*, and ROS accumulation is likely associated with heat tolerance in *TaFER* overexpression plants (Zang et al. 2017a, b, 2018). Further investigation revealed that constitutive expression of *TaPEPKR2* in wheat resulted in enhanced tolerance to both heat and dehydration stresses (Zang et al. 2018). Interestingly, the chromosomal location of this gene is close to the genomic interval of heat tolerance-related *QTL.ICD.Heat.09§* was identified by Hassouni et al. (2019) with a physical distance of ~ 2.7 Mb (Table S2). Guo et al. (2015) reported that overexpressing wheat NAC transcription factor *TaNAC2L* in *Arabidopsis* led to an increased survival rate of seedlings under heat stress conditions, and 26S proteasome is involved in the regulation of TaNAC2L protein abundance at post-transcriptional level in response to heat stress. Moreover, wheat 12-oxo-phytodienoic acid reductase (*TaOPR3*), involved in jasmonate (JA) biosynthesis, is up-regulated when facing heat stress, and its knockdown lines show enhanced heat sensitivity, whereas overexpression lines exhibit improved heat tolerance. In *Arabidopsis*, HSFA1b binds heat shock elements of AtOPR3, a homolog of TaOPR3, results in activation of AtOPR3 and JA accumulation after heat stress, indicating a mechanistic link between HSFs and JA signaling pathway in response to heat stress (Tian et al. 2020) (Fig. 1).

## Conclusions

Heat stress is a limiting factor resulting in wheat yield loss worldwide, and the occurrence of heat events is projected to increase in the future. It is estimated that yield loss and post-heading heat stress are significantly correlated, especially, when heat stress occurred together with drought stress, their interaction will highlight yield variability, explaining approximately a third (32–39%) of wheat yield loss (Ray et al. 2015). Therefore, understanding the genetic basis and molecular mechanisms of heat response will pave a way to improve heat tolerance during wheat breeding programs. Yet, this quantitative agronomic trait is controlled by multiple genes with minor effects, and probably due to huge genomic constitution, no major gene responsive to heat stress has been isolated using map-based cloning method in wheat till now, although a bunch of heat stress-related QTLs were obtained. However, with the release of wheat reference genome and the advent of state-of-art technology, map-based gene cloning is becoming easier nowadays than before in wheat. Thus, it needs more effort to go into the project of heat stress gene cloning during subsequent studies. In addition, functional analysis of heat-responsive wheat gene is often performed in model plants in previous studies, because wheat transgene technology is not reliable then. However, the situation is changed now and overexpression, RNAi and CRISPR-Cas9 technology have been widely used in wheat recently. Therefore, we propose that map-based gene cloning and molecular mechanisms of heat response gene will speed up in wheat in the future. However, we have to notice that overexpression or pyramiding of heat-responsive gene often results in side effects on crop yield according to the previous studies in model plants. How to improve wheat heat tolerance without yield penalty is an important issue we have to face. The study of the rice *TT1* gene provides us a new insight into the usage of heat-tolerant gene that the substitution of one amino acid might lead to protein conformation variation or protein stability change when subjected to heat stress, and subsequently contribute to heat tolerance (Li et al. 2015). Therefore, we should pay more attention to superior allele identification, which can both promote heat tolerance and reduce yield and quality penalty in a wheat breeding program.

## Supplementary Information

Below is the link to the electronic supplementary material.

Supplementary file1 (DOCX 53 KB)
