# Supplementary material for: The genetic and molecular basis for improving heat stress tolerance in wheat
Source: aBIOTECH. 2021 Dec 3;3(1):25–39. doi: 10.1007/s42994-021-00064-z (PMC9590529; doi:10.1007/s42994-021-00064-z)
Supplement: Supplementary file 1 — Supplementary file1 (DOCX 53 KB) [file 42994_2021_64_MOESM1_ESM.docx]

| **Table S1. Description of heat stress treatment conditions for QTL mapping** | | | | | | |
| --- | --- | --- | --- | --- | --- | --- |
|  | Normal Growing Condition | Heat Stress Conditions | Heat Stress Stage | Control | Heat Stress Characterization | Heat Stress Time |
| (Bhusal et al. 2017) | Field | Field | - | Normal sown | Late sown | - |
| (Guan et al. 2018) | Field | Field | - | Normal sown | Late sown | - |
| (Hassan et al. 2018) | Field | Field | - | Normal sown | Late sown | - |
| (Hassouni et al. 2019) | Field | Field | Time of booting (Z45) | Normal sown | Plastic tunnel | Day to dough stage(Z83) |
| (Li et al. 2019) | Field | Field | 1‐week post‐anthesis | Normal sown | Thermal stress shelters | Day to physiological maturity |
| (Mason et al. 2010) | Greenhouse, 20°C/18°C and 14/10h day/night | Chamber | 10 days after anthesis of the first pollinated spike | 20/18°C day/night | 38/18°C and 9/15h day/night | 3 days |
| (Mason et al. 2011) | Greenhouse, 20°C/18°C and 14/10h day/night | Greenhouse | 10 days after anthesis of the first pollinated spike | 20/18°C day/night | 38/18°C and 9/15h day/night | 3 days |
| (Maulana et al. 2018) | Chamber | Chamber | Three-leaf stage | 25/20°C and 16/8h day/night | 40/35ºC and 16/8h day/night | 14 days |
| (Mohammadi et al. 2008) | Greenhouse, 20-25ºC | Chamber | 1 week after the first anther extrusion was observed | 20-25ºC | 35/30ºC and 14/10 h day/night | 3 days |
| (Paliwal et al. 2012) | Field | Field | - | Normal sown | Late sown | - |
| (Sangwan et al. 2019) | Field | Field | - | Normal sown | Late sown | - |
| (Shirdelmoghanloo et al. 2016) | Greenhouse, 24/18°C day/night | Chamber | 10 days after their respective anthesis dates | 24/18°C day/night | 37/27°C day/night | 3 days |
| (Talukder et al. 2014b) | Greenhouse, 20/15 ± 2°C and 16/8h day/night | Chamber | 10 days after anthesis | 20/15 ± 1°C and 16/8h day/night | 36/30 ± 1°C and 16 /8h day/night | 10 days |
| (Telfer et al. 2021) | Greenhouse | Chamber | Reached the end of anthesis (Z69) | Normal sown | 36/16°C and 8/16h day/night | 3 days |
| (Wang et al. 2021) | Field | Field | - | Normal sown | Late sown | - |
| (Yang et al. 2002) | Chamber, 20/15°C and 16/8h day/night | Chamber | 10 days after anthesis of the second spike | None | 30/25ºC and 16/8 h day/night, | Day to physiological maturity |
| (Zhai et al. 2021) | - | - | Three-leaf stage | None | 38°C for three days followed by recovery at 20°C for three days. | 3 days |

**Table S2. Chromosomal location of functional genes in wheat**

| Trait | QTL | Marker | Chromosomes | Gene | Physical distance/Chromosome location |  |
| --- | --- | --- | --- | --- | --- | --- |
| HSI of Thousand grain weight | QHSI oftgw.cau-5B | barc59 | 5B | *TaNAC2L* | 17.2 Mbp/653,432,387-653,434,033bp | (Guan et al. 2018; Guo et al. 2015) |
| SSI of Grain yeild | QTL.ICD.Heat.09§ | AX-95182463 | 5B | *TaPEPKR2* | 2.7 Mbp/424,332,469-424,336,121bp | (Hassouni et al. 2019; Zang et al. 2018) |
| Thylakoid membrane damage | QHttmd.ksu-7A | Xbarc121 | 7A | *TaOPR3* | 8.5 Mbp/603,291,570-603,295,119bp | (Talukder et al. 2014b; Tian et al. 2020) |
| - | - | - | 4D | *TaHsfA6f* | -/447,638,791-447,641,580bp | (Bi et al. 2020) |
| - | - | - | 2A | *TaHSP23.9* | -/ 537,456,017-537,457,024bp | (Wang et al. 2020) |
| - | - | - | 7B | *TabZIP60* | -/536,004,935-536,007,205bp | (Geng et al. 2018) |
| - | - | - | 5B | *TaFER* | -/278,795,253-278,797,792bp | （Zang et al. 2017b） |
| - | - | - | 5B | *TaOEP16-2* | -/51,633,810-51,637,435bp | （Zang et al. 2017a） |
